# A commentary of “Discovery of an extremely narrow jet and 10 teraelectronvolt photons from the brightest-of-all-time gamma-ray burst”: Top 10 Scientific Advances of 2023, China

**DOI:** 10.1016/j.fmre.2024.03.011

**Published:** 2024-03-29

**Authors:** Renxin Xu

**Affiliations:** State Key Laboratory of Nuclear Physics and Technology, School of Physics, Peking University, Beijing 100871, China

In the past hundred years since 1900, with some sorts of fundamental symmetry, the combination of quantum theory and special relativity is successful in establishing the “standard model of particle physics”. In this era of multi-messenger astronomy (encompassing full electromagnetic waves, neutrinos, cosmic rays, and gravitational waves), it becomes increasingly important to explore the physics in strong gravity, which involves the integration of the standard model and the general relativity. The mystery of powerful and brief flashes of γ-rays, called gamma-ray bursts (GRBs), is one of such issues, often involving the birth of black holes and neutron stars. LHAASO (Large High Altitude Air Shower Observatory), running at full-time with a wide field of view, has luckily captured an event, called GRB 221009A, the brightest of all time (BOAT). That detection is meaningful for understanding the explosive radiation, and hence the central engine relevant to the super-dense matter in strangely curved space-time.

Scientists initially focused solely on terrestrial gamma rays emitted by radioactive elements, without considering their detection in space, until a treaty was signed by the United States, the United Kingdom, and the Soviet Union to prohibit the testing of nuclear weapons in 1963. The Vela satellites, equipped with detectors sensitive to X-ray and γ-ray flashes, were subsequently launched to ensure compliance, leading to the discovery of cosmic γ-ray bursts [1].

The key question of what caused the bursts remains unanswered, even after more than half a century of research. Nevertheless, the consensus amongst astronomers is that GRBs arise from extremely catastrophic events in distant galaxies, producing jetted fireballs with extremely relativistic particles shooting at us during the formation of compact stars. The initial jets radiate *prompt* γ-ray emission (from milliseconds to minutes), and follow-up radiation (called *afterglows*), due to the interaction with circumburst material, could usually be detected in X-rays, ultraviolet, visible, infrared, and radio bands for hours to months.

GRBs offered a surprise in 2022 when the Fermi space telescope detected and located the burst, named GRB 221009A (nicknamed the BOAT). The exceptionally large fluence of GRB 221009A saturated almost all gamma-ray detectors. Its optical afterglow was soon detected by the Very Large Telescope, showing a very red continuum with absorption features at a relatively low redshift of 0.15 (a distance about 2 × 10^9^ light-years). Fortunately, the LHAASO Collaboration observed its afterglow at a very-high-energy band, capturing both the prompt emissions and rising afterglow phases for the first time (Fig. 1).

**Fig. 1:**
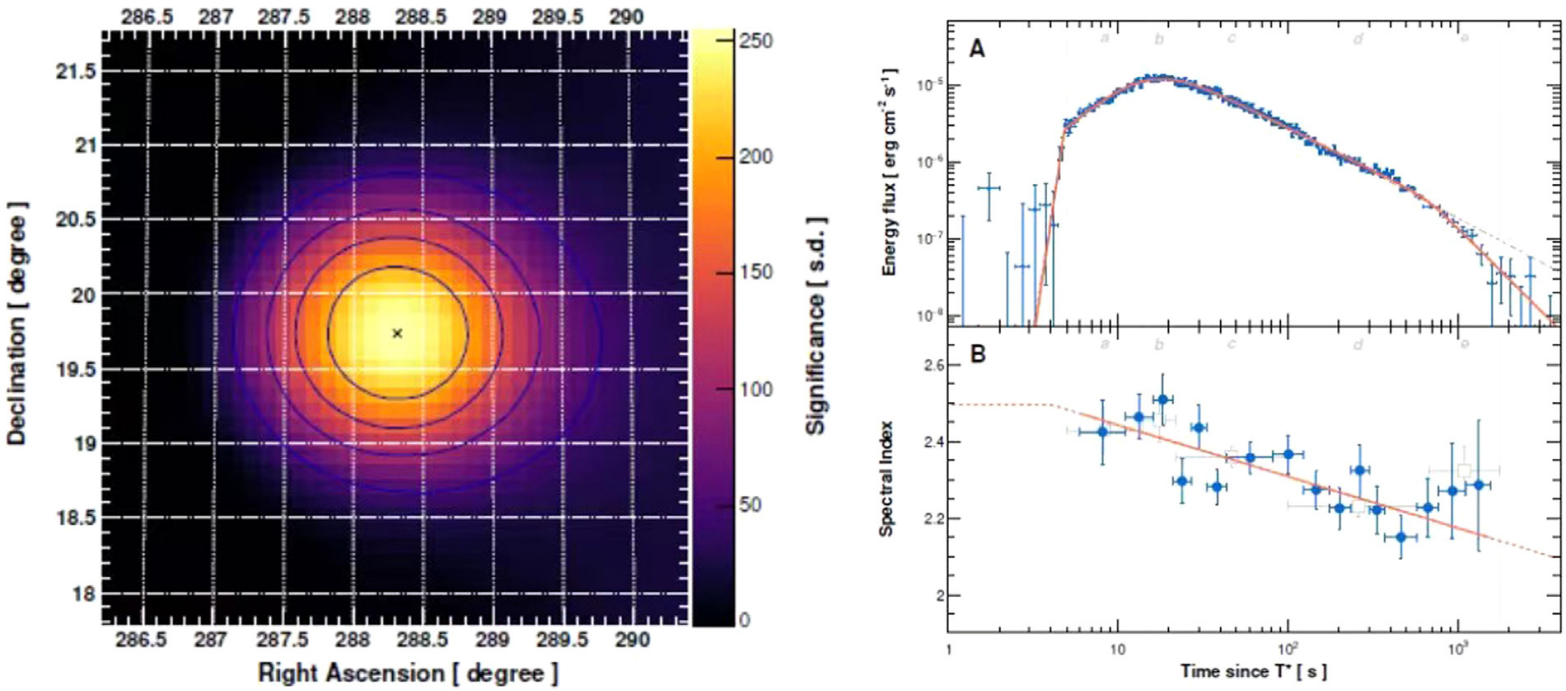
**Significance map of the GRB emissions detected by LHAASO-WCDA (Left) and energy flux light curve and spectral evolution in the VHE band for GRB 221009A (Right)**[2].

As one of China's mega-facilities, LHAASO observes the Universe through studying extensive air showers caused by the high-energy cosmic radiation including protons and photons, which, taking advantage of the high altitude at the Tibet Plateau, is peculiar in the astronomy of very-high-energy γ-rays (VHE, from 0.1 TeV to ∼PeV). With its larger instantaneous field of view and full observing duty cycle, LHAASO recorded the prompt and afterglow onset of GRB 221009A [2]. It collected more than 6 × 10^4^ photons within less than an hour, including the highest photon with 18 TeV, the microscopic photon with energy comparable to the kinematic energy of finger tapping on the keyboard! Most importantly, LHHASO measured the rise at peak but followed by a decay phase, which becomes more rapid at ∼650 s, indicating a relativistic jet with half-opening angle of only ∼0.8°. This small beamed jet, combined with the low redshift, means that the total energy of the burst of GRB 221009A during about ten minutes is around 10^51^ ergs, approximately corresponding to the total energy released by the current Sun within 10^11^ years! Yes, as the BOAT, GRB 221009A is actually usual but among the highest measured because of both the small beaming angle and its close neighbor. Promising models related to GRB central engines need to improve to reproduce this structured jet with a small angle.

The GRB study, an archetype of investigating the physics in strong gravity, is far from reaching a final solution. It becomes a common view that GRB's central engines are black holes, formed by either cataclysmic deaths of massive stars or merging neutron stars [3], but the formation of neutron stars cannot yet be ruled out [4]. Interestingly, observational evidence to support the late activities of GRB's central engines is accumulating [5,6], which favors neutron stars as the engines. It may hint at some explosive dynamics if the narrow jets like GRB 221009A are common. Certainly, the research of neutron star matter has so far proved inconclusive, which depends on the non-perturbative behavior of the fundamental strong interaction, and hopefully, GRBs may help.

## Declaration of competing interest

The author declares that he has no conflicts of interest in this work.
